# Reading English-language haiku: An eye-movement study of the ‘cut effect’

**DOI:** 10.16910/jemr.13.2.2

**Published:** 2020-01-17

**Authors:** Thomas Geyer, Franziska Günther, Hermann J. Müller, Jim Kacian, Heinrich René Liesefeld, Stella Pierides

**Affiliations:** General and Experimental Psychology LMU, Munich, Germany; Department of English and American Studies, LMU, Munich, Germany; The Haiku Foundation, Winchester, VA, USA

**Keywords:** Neuro-Cognitive Poetics, English-language Haiku, cut effect, fixations

## Abstract

The current study, set within the larger enterprise of Neuro-Cognitive Poetics, was designed to examine how readers deal with the ‘cut’ – a more or less sharp semantic-conceptual break – in normative, three-line English-language haiku poems (ELH). Readers were presented with three-line haiku that consisted of two (seemingly) disparate parts, a (two-line) ‘phrase’ image and a one-line ‘fragment’ image, in order to determine how they process the conceptual gap between these images when constructing the poem’s meaning – as reflected in their patterns of reading eye movements. In addition to replicating the basic ‘cut effect’, i.e., the extended fixation dwell time on the fragment line relative to the other lines, the present study examined (a) how this effect is influenced by whether the cut is purely implicit or explicitly marked by punctuation, and (b) whether the effect pattern could be delineated against a control condition of ‘uncut’, one-image haiku. For ‘cut’ vs. ‘uncut’ haiku, the results revealed the distribution of fixations across the poems to be modulated by the position of the cut (after line 1 vs. after line 2), the presence vs. absence of a cut marker, and the semanticconceptual distance between the two images (context–action vs. juxtaposition haiku). These formal-structural and conceptual-semantic properties were associated with systematic changes in how individual poem lines were scanned at first reading and then (selectively) re-sampled in second- and third-pass reading to construct and check global meaning. No such effects were found for one-image (control) haiku. We attribute this pattern to the operation of different meaning resolution processes during the comprehension of two-image haiku, which are invoked by both form- and meaning-related features of the poems.

## Introduction

The aim of the present study was to follow up on a prior, more exploratory investigation (1) of the reading of standard (i.e., three-line) two-image English-language haiku (ELH) of the ‘context–action’ and ‘juxtaposition’ types (2; see also below). Our previous study provided some intriguing indications, or hypotheses, from the analysis of eye movements during reading, of how the two images put into a (more or less) tense relation in such haiku might be aligned in a process of global meaning construction. However, firm conclusions were limited as the various types and structural properties of the ELH presented for reading were not perfectly balanced and the study design did not include a control condition against which to compare the reading of the two-image haiku. These limitations were overcome in the present, more controlled study. The results both confirm and, in critical ways, extend our previous findings.

To set the stage, we first provide the study background within the larger enterprise of (Neuro-)Cognitive Poetics (we bracket the ‘Neuro-’ in Neuro-Cognitive Poetics because our approach in the present study is mainly ‘cognitive’) and describe why ELH are a particularly interesting study material, before reviewing our previous findings and developing the questions at issue in detail.

### (Neuro-)Cognitive Poetics

Reading is a central activity in our everyday life. We are continuously encountering an increasingly complex – sensory, social, economic, etc. – environment, where reading can help us obtain the information necessary for reducing uncertainty and can thus guide decision-making. Since “[i]t seems psychologically unlikely that we have developed different cognitive strategies for dealing with fictional worlds and non-fictional worlds” (3, p. 92), it can be assumed that immersion in the world of literary writing (novels, poetry, etc.) also opens up a space for learning – from a world of images, symbols, and stories – by (re-) creating/simulating fictional worlds, situations and actions in our minds. Such (cognitive and embodied) processes can centrally contribute to enriching our capacity for empathy, imagination and understanding (4, 5, 6), for example, by fostering readers’ ability of identifying and understanding other people’s mental states (i.e., by enhancing their *Theory of Mind*; van Kuijk et al., 2018) and by functioning as triggers of experiences of insight (7) and aesthetic appreciation (8, 9).

In this respect, the latter effects inform our shared consciousness and humanity. They enable us to experience, for instance, a sense of unity and wholeness, simple as well as revelatory, in moments of insight, such as when a wildflower opens up to us with all its completeness and beauty. Writers and poets attempt to share this experience by recreating it in the mind of the reader (see e.g., 10, for the different approaches to writing haiku). How this may be achieved, what processes of (re-)construction and insight go on in the reader’s mind (and brain), is a question that has concerned poets for a long time, with a view to utilizing this knowledge in their work. For instance, Matsuo Bashō, the 17th-century Japanese haiku master, gave this advice to haiku poets: *“Go to the pine if you want to learn about the pine, or to the bamboo if you want to learn about the bamboo. And in doing so, you must leave your subjective preoccupation with your self. Otherwise you impose yourself on the object and do not learn. Your poetry issues of its own accord when you and the object have become one—when you have plunged deep enough into the object to see something like a hidden glimmering there. However well phrased your poetry may be, if your feeling is not natural—if the object and yourself are separate— then your poetry is not true poetry but merely your subjective counterfeit”* (cited in: Kacian, 2006, pp. 42–43). More recently, this question has attracted the interest of researchers in the areas of Cognitive and Neuro-Cognitive Poetics (3, 11, 12, 13, 14, 15).

Central to this field of (Neuro-)Cognitive Poetics is the idea that studying the processing of literary language – in particular poetry – provides an apt approach for bringing together the cognitive with the emotional level of processing (16). The cognitive perspective focuses on how the understanding of literary texts is achieved conceptually (e.g., via processes like (real and simulated) perception, thought, conceptualization, prediction/expectation generation, etc.), that is, it emphasizes “rational decision-making and creative meaning construction” ( 3, p. 151). The complementary, emotional perspective focuses on the motives and feelings intricately involved in (i.e., both driving and associated with) the comprehension processes. Among those affective components of literary reading count, for instance, thrill and pleasure-seeking, experiences of joy or sadness, as well as aesthetic liking and appreciation. The aim of (Neuro-)Cognitive Poetics is thus to bring together the cognitive and affective perspectives in one account of literary reading (16, 17, 18).

This dual approach has perhaps been spelled out most systematically by Jacobs and collaborators. In their (qualitative) model of literary reading (16, 17), they assume that all literary texts, including even single words in isolation, consist of, and transport, background [BG] and foreground [FG] features, in various mixture ratios (see also 6) 1. When combined, these elements constitute the ‘meaning Gestalt’ of a text (19). Gestalt Psychology ( 20, 21, 22, 23) has described processes that organize the array of elementary features in the visual field into unified, coherent ‘objects’ that can become the focus of attention in perception (against a background ‘context’). In analogy to the Gestalt principles of perceptual organization (for applications in cognitive linguistics, see, e.g., 24, 25, 26), processes of literary construction and appreciation are seen as encouraging play with different perspectives, conceptions, and expectations, and thus of processes that are all directed towards eventually arriving at a coherent, contextualized ‘meaning Gestalt’ for a text.

According to Jacobs (16), shifts between background and foreground features figure centrally in this process of literary comprehension (see also 8). BG features are said to be the elements of a text that create a feeling of familiarity in the reader: familiar words, phrases, and images; familiar situation models, socio-cultural codes, and affective scripts. As such, BG features are coherent with readers’ previous experiences and expectations, and thus provide them with a context against which the cognitively more challenging FG features stand out and in which they can be grounded (3). Background features therefore enable rich and relatively effortless cognitive simulation, and, accordingly, facilitate automatic (fast) processing of the respective passages of literary texts (16, 17). In contrast, FG features, such as unusual form elements (including, in poetry, the use of line breaks) and semantic vagueness or ambiguity as well as textual inconsistency or (seeming) incoherence, may be brought in a relationship of tension or conflict with the BG elements, interrupting the (automatic) processing of texts by capturing attention.

In such situations, the repertory of standard cognitive and affective schemas no longer suffices to make meaning. Instead, FG elements challenge the situation model that a reader has formed on the basis of the BG elements and make it necessary for her/him to reconsider and update this model. This will trigger a controlled (slow) reading mode, involving ongoing, cognitively challenging processes of ‘meaning Gestalt’-construction through information integration and synthesis. Reaching the end of this effortful “aesthetic trajectory” (27) is likely experienced as rewarding: “after initial moments of familiar recognition, followed by surprise, ambiguity, and tension, the closure of meaning gestalts [releases the tension and is] … occasionally supplemented by an ‘aha’ experience … or feeling of good fit, ‘rightness’, or harmony …” (16, p. 16).

### Haiku as paradigmatic study material for (Neuro-)Cognitive Poetics

**Figure 1.**
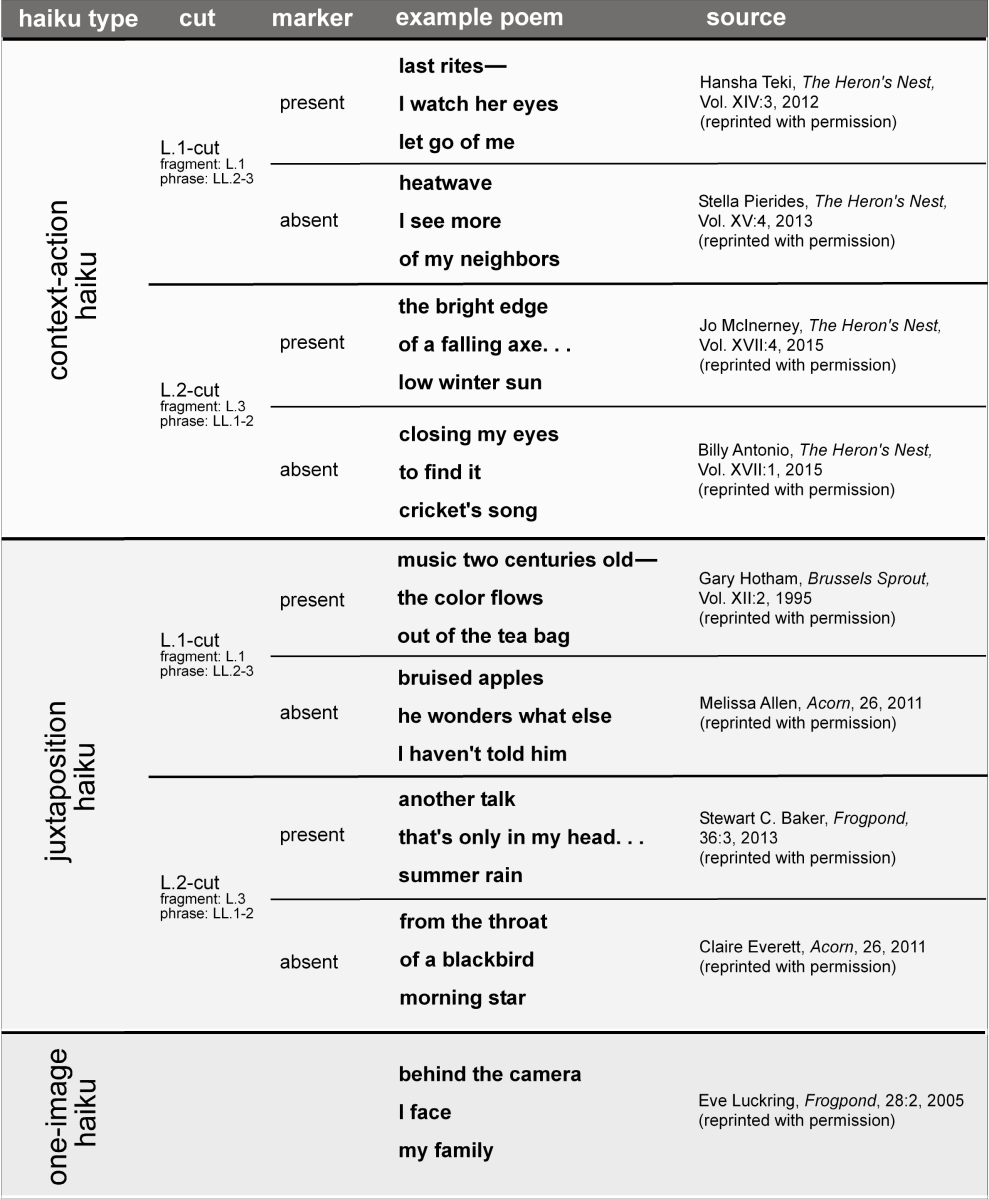
Example haiku from the sample used in the study, for each of the eight haiku type × cut position × cut marker conditions. In context–action haiku, one component (image) of the haiku, the fragment, provides the context (take, for example Hansha Teki’s, 2012, poem: [fragment] “last rites–“) and the other, the phrase, describes an action set within this context ([phrase] “I watch her eyes / let go of me”). Both images, although each relatively familiar, are set in a relationship with one another by the poet. In juxtaposition haiku, by contrast, there is no straightforward (familiar) context–action relationship, that is, the images juxtaposed are more jarring, in a relationship of tension that needs to be resolved (e.g., Melissa Allen’s, 2011: [fragment] ”bruised apples /” [phrase] “he wonders / what else I haven’t told him”). The cut can either be at the end of line 1 (L.1-cut, i.e., the fragment part is in line 1) or at the end of line 2 (L.2-cut, i.e., the fragment is in line 3). Further, cut effects can be reinforced by punctuation (i.e., explicit cut markers) at the end of the fragment line 1 (L.1-cut haiku) or central line 2 (L.2-cut haiku). In the present study, reading behavior was also assessed in a control condition: one-image haiku (e.g., “behind the camera / I face / my family”, Eve Luckring, 2005) with only a single picture/image, i.e., without tension between conflicting background and foreground elements (fragment and phrase lines in haiku, respectively).

In the (Neuro-)Cognitive Poetics approach, texts are analyzed and used for investigating the cognitive and emotional processes involved in their reception (8, 28, 29). In a recent study (1; see also, 30, 31), we argued that short forms of poetry, and in particular the specific form of normative, three-line ELH (32), provide a ‘paradigmatic’ material for studying the reading of poetic texts (another type of short poetry worth considering in future research might be *microrrelatos*; see, e.g., Lagmanovich, 2006). Haiku poems (see Figure 1 for examples) record a moment of insight into the nature of the world, in an effort to share it with others (2). The contemporary poet aims to convey her/his experience of that moment (including recollected as well as imagined moments) in the present, in words that render it so concisely and directly – without commenting, explaining, or marveling at the experience – and, at the same time, so suggestively – making the words expand in the reader’s mind into a multitude of images and feelings – that it is possible for the reader to re-create and share that moment and the experience it encapsulates.

Normative ELH – brief poems, unrhymed, unfolding over three lines, in a short–long–short line pattern, with, as a rule, fewer than 17 syllables in total – fulfill two desiderata for empirical studies: (i) While varying widely in content (meaning), they are compositionally well constrained and highly similar in structure; they thus constitute ideal test material for experimental research in (Neuro-)Cognitive Poetics by allowing for systematic variation of stimulus features and repeated measurement. (ii) ELH engage a rich set of mental functions with the minimum of linguistic means, using everyday, unadorned language, characterized by the use of high-frequency vocabulary and ‘plain style’ ( 10), thus offering a potent literary form for investigating processes of meaning construction. Importantly, processes of arriving at a coherent ‘meaning Gestalt’ (19) can be assumed to figure centrally in ELH comprehension, since it requires the resolution of surprise induced by the fact that ELH usually juxtapose two seemingly unconnected images.

It is this clearly defined design feature of image juxtaposition, and the consequent need of resolving the tension between the two images at the core of ELH, which renders them a particularly suitable study material for (Neuro-)Cognitive Poetics: it confronts readers with a particular, genre-specific pattern of BG–FG alternations, which will be described in more detail in the following.

Contemporary haiku poets use ordinary, everyday words, images, and concepts, importantly including keywords (such as *cherry blossom, harvest moon, or new year’s eve*) that refer to a season, occasion, or aspect of the environment and have a rich, and long, tradition known to, and shared by, the poets and their (initiated) readership. Such keywords thus evoke in the reader’s mind, in a nutshell, a season of the year and associations, literary connections, and (partly cultural) scripts that ground the poem. Accordingly, they provide background features that allow for an element of immersion on the part of the reader. As their characteristic foregrounding element, normative two-image haiku contain a ‘cut’ (referred to as ‘ *kire*’ in the Japanese tradition), that is: a break point or gap between two (at first glance) often seemingly disparate images, or parts, of the haiku. ELH thus make central use of the poetic device of juxtaposition: two images (2) – or, in Reichhold’s (33) terms, *fragment and phrase* – are juxtaposed side by side in a more or less tense relationship, inviting comparison of the haiku’s constituent elements to unravel the significance of the moment the poet presents, that is, to (re-)construct the poem’s meaning (2). Haiku poets consider the kire or cut as the central feature of haiku and the engine by which it runs; and the gap created by the cut is crossed by the charge of meaning(s) which seek to unify the poem – a successful haiku is one which completes the circuit in a unique and unexpected, yet totally satisfying, way.

Structurally, the cut may be placed either at the end of the first line (i.e., the first line constitutes the fragment and lines 2 and 3 the phrase; henceforth referred to as *L.1-cut haiku*) or the end of the second line (i.e., lines 1 and 2 constitute the phrase and line 3 the fragment; henceforth *L.2-cut haiku*) – for examples, see Figure 1. Conceptually, the strength of the juxtaposition (the semantic distance) between the fragment and phrase images varies between different types of haiku. In *context–action haiku*, “one of the images … establishes the setting where the haiku moment is experienced; the other suggests the activity which caught the notice of the poet’s imagination” – so, for the reader, closing the gap between the two images is more straightforward. In *juxtaposition haiku*, by contrast, “two images not obviously related by context or action are paired” (2) – with a clear, recognizable break, or gap, between the two parts.

With both types of haiku, the cut gives rise, at first, to a startling experience and feelings of discrepancy, which in turn activates processes of reflection and re-appraisal to bridge the gap and close the haiku’s meaning. The realization of how the juxtaposed images fit together is referred to as *haiku moment*, which may involve an ‘aha’ experience, aesthetic appreciation, and feelings of reward. By invoking this aesthetic trajectory, haiku thus invite reader participation in (re-)constructing the poem’s meaning and experiencing the haiku moment (see, e.g., 10, for an extended discussion of different approaches to haiku writing and reading).

On this basis, we proposed that “haiku provide an ideal study medium for neuro-/ cognitive poetics: the constructive device of juxtaposition, within the context of the brevity and compositional consistency of the form, makes haiku highly attractive for the scientific investigation of central processes that go on in the reader’s mind-brain while reading and appreciating poetic texts” (1, p. 6).

### The ‘cut’ effect in our first study

The aim of our initial study (1) was to explore some of the processes involved in ELH reading – in particular: processes involved in dealing with the cut – by means of recording and analyzing the eye-movement patterns participants produce while reading and re-reading haiku. This approach is based on the ‘eye-mind (immediacy) assumption’ (34): eye movements tell us where, when, and for how long attention is allocated within the text to extract information and integrate it into global meaning. The question was whether cut position effects (and their potential modulation by haiku type) would *at all* be reflected (or be discernible) in the eye-movement patterns.

In some of its aspects, our study thus adds to the existing tradition of investigating the effects of violations of semantic and/or discourse coherence on eye-movement patterns in text reading in general (e.g., Camblin et al., 2007; van Der Schoot et al., 2012; Wang et al., 2008), and in the reading of specific text types (e.g., jokes: Ferstl et al., 2017, or sarcastic texts: Olkoniemi et al., 2019) in particular. However, our approach goes beyond this tradition in that the cut effect in haiku, rather than being exclusively driven by semantic incoherence, is brought about by the unique combination of patterns of (seeming) semantic incoherence with genre-specific syntactic and form features.

Although partly in line with recent investigations of genre-specific eye-movement effects – such as in multimodal texts like comics or graphic novels (e.g., Laubrock, Hohenstein & Kümmerer, 2018) –, demonstrating characteristic eye-movement effects of such a multidimensional device of genre-specific poetic writing as the ‘cut’ would, to the best of our knowledge, have novelty value in the cognitive-poetics literature. While some stylistic and form features typical of poetic texts, like the spatial layout of the text on the page ( 35) or the stylistic device of enjambment (36, see also 37) have been identified to have specific effects on eye movements during reading, to our knowledge, there have not been other findings of signature eye-movement patterns reflecting the more content-related features of an unexpected sharp thematic or imagistic ‘turn’ in poetry, as is, for instance, also characteristic of sonnets (38).

The most striking finding in our initial study of haiku reading was a marked *cut effect*: fixational dwell times (aggregated fixation durations per word, normalized per words in a line) were longer in the haiku’s fragment line than in the phrase lines. We took this to suggest that encountering the cut acts as a foregrounding, attention-capturing feature, with the eye and thus the mind then focusing predominantly on the fragment line, which provides the ‘pivot’ for meaning resolution (through the establishment of textual coherence via the integration of the two images). This cut effect was evident both when the cut occurred at the end of line 1 (L.1-cut haiku: fragment in line 1) and when it occurred at the end of the line 2 (L.2-cut haiku: fragment in line 3), though it was more marked in the latter case. Also, the cut effect was stronger for juxtaposition than for context–action haiku, reflecting the strength of the (functional-)conceptual distance or discrepancy between the two parts. Accordingly, the fact that we were able to establish such signature eye-movement patterns when readers (who, in our exploratory study, were naïve to the genre) encounter the cut in haiku suggests that normative ELH are a particularly potent material for studying on-line processes of literary meaning construction in (Neuro-)Cognitive Poetics.

Thus, our exploratory study provided promising evidence of the cut effect (expressed in signature eye-movement patterns) in the reading of haiku. However, there were several methodological caveats – relating to the presence/absence of explicit cut markers (i.e., punctuation marks; see below for details), imperfectly balanced cut position and haiku type samples, and lack of a control condition – that limited any firm conclusions.

Concerning cut markers: haiku poets may indicate, and emphasize, the cut by the use of explicit punctuation, such as dash (‘—’), ellipsis (‘…’), comma, semi-colon, question mark, etc. (example: “last rites– / I watch her eyes / let go of me”: 39), whereas in others the cut is an unmarked, text-inherent feature (example: “bruised apples / he wonders / what else I haven’t told him”: 40). The use of cut markers is not obligatory, but rather a matter of poetic choice or technique: the cut itself would normally be clearly discernible even without the use of markers (41). On the part of the reader, while encountering a cut marker may initially give rise to a startling experience, interrupting the flow of reading, at least certain types of punctuation suggest a certain ‘reading’, thus guiding the integration of the juxtaposed images. For instance, an ellipsis marker will prompt the reader to think beyond what is being said, about what is being omitted or alluded to, which may lead to the development of expectations that inform the reading of the post-cut line(s); similarly, a question mark may prompt the reader to generate possible (likely) answers, which then again inform further meaning construction, while a dash may indicate a pause like a full stop, and strongly imply the introduction of new, unexpected material. That is, cut markers might trigger processes of active (semantic/episodic) memory search and the formation of reader expectations, which can then function as top-down constraints on the reading of the remaining text. While such additional processes would be effortful and consume time, engaging in them may ultimately yield savings, because the markers – which were placed there very deliberately by the poet – provide pointers to the poem’s meaning and prompt readers to engage in processes required for meaning resolution relatively early in reading.

These predictions concerning possible effects of cut markers are largely in line with findings from studies of general effects of punctuation (mostly commas) in (non-literary) reading. Those report that punctuation marks correlate with reduced reading speed/‘pauses’ before the mark as well as facilitated processing of the text passage immediately following it, including the reduction/prevention of regressive movements (42). This might, generally, indicate that they function as markers of higher-level/functional processing units in reading (43). More specifically, it has been suggested that (a) punctuation marks might constitute triggers for meaning wrap-up processes (42), as well as for the (related) generation of expectations on the subsequent passages (44); that they (b) might constrain the scope of certain modes of processing (e.g., distributed as opposed to serial processing; see: 43). In addition, there is (c) evidence that punctuation marks play an important role in how a clause is parsed and, consequently, interpreted. Several studies find that punctuation marks suggest one pattern of resolution for a structurally ambiguous clause or sentence more strongly than its possible (competing) alternatives (45, 46), and thus contribute centrally to processes of disambiguation (47, 48). Furthermore, some findings suggest that which particular kind of punctuation mark is used can make a difference (45). This aspect, however, has received relatively little attention in research so far. The same accounts for context-, condition-, and genre-specific effects ( 42).

Thus, at least when a marker is present to emphasize the cut in a haiku, it may not be surprising that a cut effect is actually observed. However, in our exploratory study, the presence versus absence of explicit cut markers was an uncontrolled variable, and so we could not be certain whether a cut effect would arise even in haiku without explicit markers, or to what extent our effect pattern was attributable to more formal – rather than content-based – properties of our reading material. Second, our sample poems presented for reading were not entirely balanced (e.g., they included only relatively few L.2-cut context–action haiku, which appear to be overall rarer in the literature). This left open the possibility that the (structural, semantic) specifics of the particular poems that we presented in the various conditions (cut position: L.1-cut vs. L.2-cut × haiku type: context–action vs. juxtaposition), especially conditions with fewer poems, may have driven the more complex, interaction effects (i.e., the cut effect being modulated by cut position and haiku type). Third, our exploratory study lacked a control condition against which to compare the reading of our cut, two-image haiku – see our discussion of the limitations in the previous paper (1).

### Objectives and overview of the present study

Given these limitations, the present study was designed to replicate, and extend, the results of our exploratory study, importantly controlling for the three problems outlined above. In particular, we adopted a fully balanced, factorial design with both structural and conceptual haiku features as independent variables. More specifically, the independent variables were: (1) (semantic) *haiku type*: context–action vs. juxtaposition, (2) (structural) *cut position*: L.1-cut vs. L.2-cut, and (3) *cut marker*: present vs. absent. We ensured equal numbers of poems in each of these (2 × 2 × 2 =) 8 conditions/categories. Also (as already in the exploratory study), the various categories were matched for a host of linguistic parameters (see full list in the Methods section below and in the Appendix) to ensure that any differential eye-movement patterns could not be attributed to (more general) linguistic factors, that is, differences that are not characteristic/definitional of the different haiku types compared.

Finally, we also introduced a control condition for our two-image haiku, that is, for the haiku with a salient cut. The question of what constitutes an appropriate control text for a poetic text is a difficult one, and our choice of control texts requires some justification. One option would have been to use some short, ‘ordinary’ text/sentence. However, it is known that approaching a text in a ‘poetic’ attitude of reading (having been instructed that the texts are poems) differs fundamentally from the reading of ordinary text (37, 49, 50, 51). An alternative control might have been a syntactically regularized, ‘uncut’ sentence (without line breaks) re-describing a haiku using (much) the same words (e.g., “As they cross the border at night, the elephant calf holds his mother’s tail”). However, such re-descriptions would not always be possible (especially for juxtaposition haiku) because the haiku’s juxtaposed parts may ‘refuse’ to be brought together in a regular English sentence – quite apart from the fact that in most cases such sentences would require the use of relatively more function words (e.g., prepositions, determiners, conjunctions, etc.), which would result in the loss of the brevity and punchiness characteristic of haiku. Merely removing the line breaks while retaining the irregular and/or fragmentary syntactic structure does not constitute an option either. As reported by Yaron (51) such poem-based texts are usually rejected by readers as unacceptable and/or incomprehensible, because they do no trigger the specific mode of ‘poetic’ reading which renders readers willing to accept and deal with seemingly obscure, formally and/or semantically highly irregular forms of language use. Thus, given that such texts are ruled out too, we opted for a ‘poetic’ control text: three-line *one-image* haiku.

As the label implies, one-image haiku render only one image (rather than two images) and are thus, by definition, ‘uncut’ (an example would be: “behind the camera / I face / my family”: 52). At the same time, they belong to the poetic genre of normative haiku and are thus characterized by similar features (such as brevity, unadorned language, and a three-line structure) as ‘cut’, two-image haiku. Accordingly, we believe that one-image haiku serve as the most suitable control condition for studying cut effects compared to the alternatives considered above. The one-image haiku we used as controls had (in our experts’ assessment) no salient cut, that is: they did not involve a juxtaposition of two semantically/conceptually as well as contextually distant images. Accordingly, arriving at a coherent interpretation of these poems should be considerably easier compared to two-image haiku, and we expected this to be reflected in the absence of the ‘cut effect’ in the reading patterns.

Note that, in addition to the recording of participants’ eye movements while they read a set of poems, they had to answer a number of subjective (rating) questions after each haiku (e.g., understanding achieved, etc.). In a later phase, participants were again presented with the poems they had read, along with a set of new poems, in randomized order, and they were required to give a recognition response (haiku already read/not read). Finally, in addition to measuring eye movements, we also recorded the electroencephalogram (EEG) during haiku reading. This produced a rich set of – eye-movement, EEG, subjective haiku rating, and recognition memory – data that will be presented in a number of papers. As for the present paper, the focus is on a closer examination of the cut effect, that is, replication and extension of the eye-movement pattern that we observed in the reading of two-image haiku in our previous (exploratory) study.

To foreshadow the outcome of the experiment reported in the following: essentially, we were able (a) to replicate the cut effects for two-image haiku; (b) to delineate them from our one-image control; and (c) to show that the use of explicit cut markers enhances these effects.

## Method

### Participants

Twenty-four participants, all students (of various academic subjects) at LMU Munich, took part in the study. Three participants had to be excluded because of technical problems during eye-tracking: poor calibration (2 participants) and loss of some 40% of data (1 participant). The remaining sample consisted of 21 participants (13 female; mean age: 25.19 years; age range: 20–36 years). They were all native speakers of English, who reported that they used English in over 80% (mean = 86.86%) of their current daily language use and that they had not started to learn any other language until after age of 7.71 years, on average. They all had normal or corrected-to-normal vision, including normal color vision. Almost all were naïve with respect to the purposes of the study (only two had participated in a previous haiku reading study). Eight participants reported to be regular readers of poetry, and two of these were experienced with haiku in particular. Participants gave informed, written consent prior to commencing the experiment and were paid at a rate of 9.00 € per hour.

### Ethics statement

The study was conducted at the Department of Psychology, LMU Munich. All experimental procedures were standard: they consisted of the collection of mainly behavioral data (eye-movement record, EEG record, subjective ratings, memory test), without involving any invasive or potentially dangerous methods, and were approved by the LMU Psychology Department’s Ethics Committee in accordance with the Code of Ethics of the World Medical Association (Declaration of Helsinki). Data were stored and analyzed anonymously.

### Apparatus

The experiment was conducted in a dimly lit and sound-attenuated chamber. It was computer-controlled (standard Intel PC, running XP operating system), with control software purpose-written in C++. Stimuli were presented on a 21-inch CRT monitor (AOC Amsterdam, NL; frame rate: 120 Hz; screen resolution: 1024 x 768 pixels). Participants sat in a comfortable armchair and viewed the monitor from a distance of 68 cm. Observers were encouraged to keep their heads still during reading (but no specific devices were used for head stabilization), in an attempt to minimize head movements (which could have compromised the eye-movement record) and muscle artifacts associated with head movements or muscle tension caused by a chin rest (which could have compromised the EEG record). Note that the current article exclusively focuses on the eye-movement data and the subjective ratings (memory-test performance and EEG effects will be reported in forthcoming papers).

**Figure 2.**
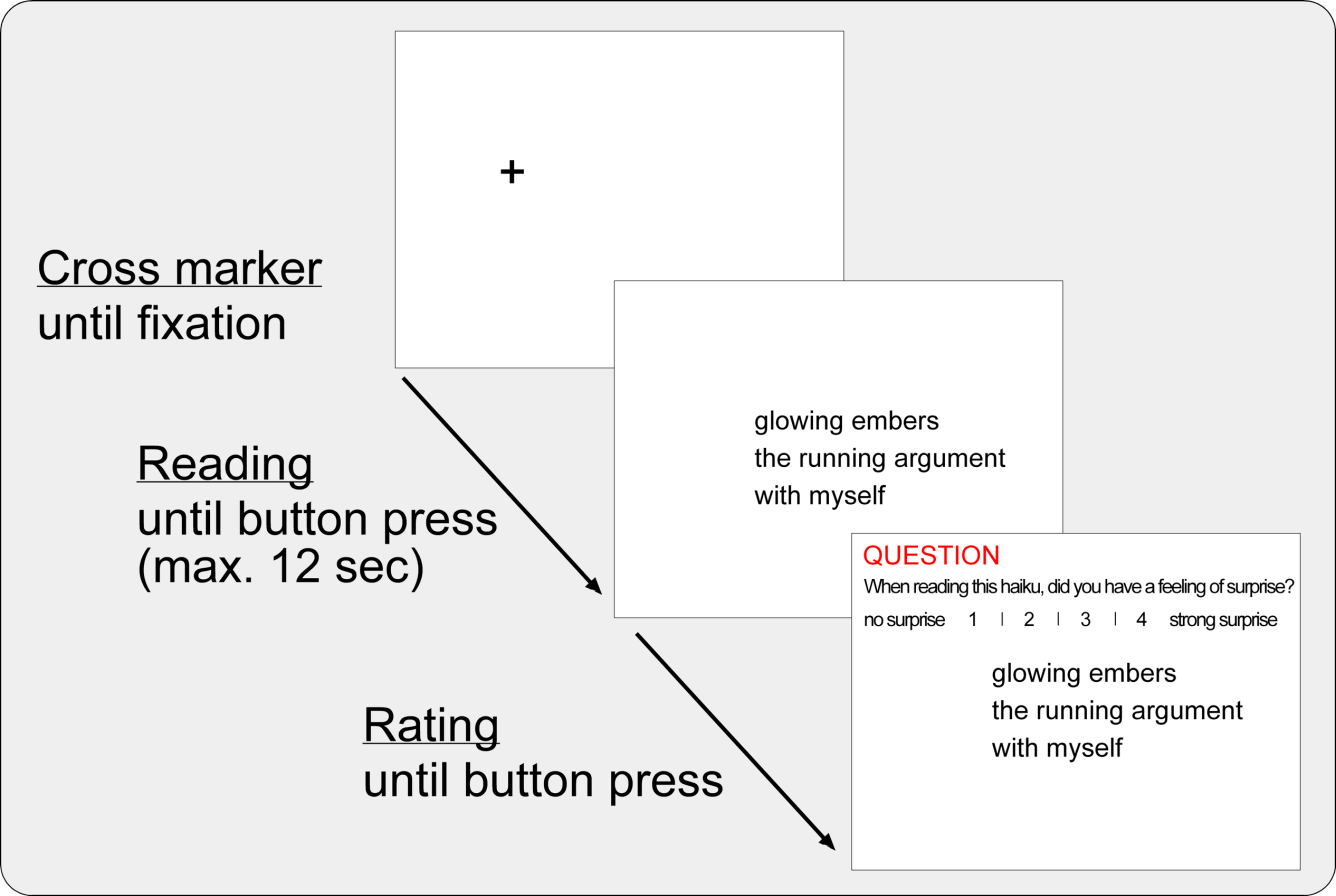
Illustration of display screens with initial fixation-cross marker (screen 1), the haiku to be read (screen 2), and an example rating question following the reading (screen 3). The poem depicted is by Stella Pierides (Pierides, 2015).

The haiku to be read during the initial reading phase of the study, all consisting of three lines, were presented left-aligned in the center of the monitor (black on white background). Prior to the onset of the haiku on a given trial, participants were presented with a black (RGB = 255, 255, 255) fixation marker, a cross symbol, to the left (i.e., the left-side boundary) of the first word in line 1; the distance between the cross and first word was 0.8° of visual angle. Overall, given the viewing distance of 68 cm (and a 21-inch screen size), the average haiku covered a screen area of some 4.4° × 8.6° of visual angle (the vertical distance between individual lines was .98°). See Figure 2 for an example display screen.

During reading, participants’ eye movements were recorded, at a sampling rate of 1000 Hz, using a remote SR Research EyeLink 1000 desktop-mount eye-tracker (SR Research Ltd., Mississauga, Ontario, Canada). Eye-movement recording was calibrated at the start of the experiment and after each 12th reading trial. Calibration was considered accurate when fixation positions fell within ~1.0° for all calibration points. Calibration was further checked at the start of each trial by the experimenter (by pressing the space key on a standard German keyboard on the control computer) as soon as stable fixation on the fixation marker was established, and ended either once the participant indicated (by pressing either the <1>, <2>, <3>, or <4> key on the numeric keyboard) that s/he had completed reading or else after the maximum haiku reading (=presentation) time of 12 sec.

Following the reading of each individual haiku, participants were presented with a set of (seven) 4-point rating scales, both to ensure the immediacy of the subjective response to poem just read and to reinforce the instruction to read with the aim of ‘understanding’ and ‘appreciating’ the haiku (see also, 53). As illustrated in Figure 2, the rating questions and scales (in the example: that for ‘surprise’) were presented above the poem. In detail, poems were to be rated in terms of: (1) “How well would you say you understood this haiku?” (scale: 1=did not understand – 4=understand fully); (2) “When reading this haiku, did you have a feeling of surprise?” (scale: 1=no surprise – 4=strong surprise); (3) “Did you experience a sudden insight into what the haiku means; i.e., did you have an 'aha' sensation?” (scale: 1=no ‘aha’– 4=strong ‘aha’); (4) “When reading this haiku, did you feel a more joyful or a more sad emotion?” (scale: 1=more sad – 4=more joyful); (5) “How strong was your feeling?” (scale: 1=very weak – 4=very strong); (6) “How beautiful or aesthetically appealing would you say this haiku is (as a short poem)?” (scale: 1=not at all appealing – 4=very appealing); (7) “How much do you like it?” (scale: 1=I do not like it – 4=I like it very much). Rating scales belonging to different cognitive/emotional ‘categories’ (understanding achieved, surprise/‘aha’, emotion valence/arousal, and aesthetic appeal/liking) were presented in immediate succession, but the category order was randomized across trials/poems (see Fig. 2).

The reading phase was followed by a memory-test phase, in which participants were again presented with the full set of haiku on the screen (those read as well as unread ‘foils’). To each haiku they had to issue (i) a yes/no recognition response and (ii), in case of a positive response, a 4-point scale rating of the certainty associated with this response.

### Materials

The ELH to be read by the participants, 64 haiku in total, and the foils additionally presented during the memory test (another 32 haiku), were selected from highly reputed (English-language) haiku journals and registries (such as *Frogpond, Modern Haiku, The Heron’s Nest, A Hundred Gourds, The Haiku Foundation*, among others). All selected poems were three-line haiku, and (apart from the one-image control haiku; see below) half of the poems were context–action haiku and half juxtaposition haiku. See Figure 1 for examples. Further, all haiku (except for the one-image controls) had a clearly discernible cut (with the two images being related by a context–action- or a juxtaposition-type relationship), either at the end of line 1 (L.1-cut haiku) or at the end of line 2 (L.2-cut haiku). In each half of the poems within each category, the cut was either unmarked or it was rendered explicit by a punctuation mark at the end of the cut line. This resulted in 2 (type of poem: context–action vs. juxtaposition) × 2 (placement of cut: L.1- vs. L.2-cut haiku) × 2 (cut marker: present vs. absent) = 8 sets of haiku or experimental conditions (with each 8 haiku per condition). In addition, there were 12 one-image haiku (8 presented during reading and another 4 during the memory test; see Fig. 1) that had no salient cut (as agreed by our haiku experts) and thus served as control stimuli.

Of note, we ensured that all 9 experimental conditions (8 two-image haiku plus 1 one-image haiku) were balanced in terms of 13 general, ‘haiku-unspecific’ linguistic parameters (item-length and frequency-related parameters, as well as selected salience-associated categorial, constructional, and stylistic features), which – based on the existing reading literature – could be expected to systematically influence attentional patterning in reading. A detailed list of these parameters and an account of the related analyses can be found in the Appendix. Given the absence of any marked differences with respect to these parameters, both among the two-image ELH in the various experimental conditions and between the two-image and one-image ELH, it is unlikely that any of the effects reported below on readers’ eye-movement behavior are mainly/primarily attributable to differences in general, haiku-unspecific language variables.

### Design and procedure

The experiment varied three (main) variables in an orthogonal manner: type of haiku (context–action, juxtaposition), cut position (L.1-cut, L.2-cut), and cut marker (present, absent), and included both the (target) two-image ELH and the one-image controls. Following an initial instruction, the experiment consisted of three distinct phases: (i) practice, (ii) reading, and (iii) memory test.

The experiment started with a practice session of a total of six trials, to familiarize observers with the reading material, the eye-tracking device, and the scheduling of events on a given trial. Only one-image haiku were shown during the practice session; these (one-image) poems were not re-presented in the subsequent reading (or memory) phase. Upon participants signaling the end of their reading by a button press, or after a maximum poem presentation time of 12 sec, they answered seven rating questions (with question order randomized across trials). The next trial started after completion of the ratings. Eye-tracking was already used during the practice trials (data not recorded) for participants to become familiar with the calibration procedure and the scheduling of events on a given trial (specifically, with having to initially hold their gaze stable at the fixation cross at trial start for the presentation of the poem to be launched).

In the reading phase, the same set of 72 haiku (32 context–action, 32 juxtaposition, and 8 one-image haiku) were presented to all participants in a trial order determined randomly for each participant. Each haiku was presented for a maximum time of 12 sec, or shorter if the participant terminated reading earlier (by pressing the <1>, <2>, <3>, or <4> key). After participants had completed reading the haiku, a set of seven rating questions was administered, asking them to indicate their understanding achieved, any experience of surprise/‘aha’ associated with reading, how they judged the emotional valence/arousal of the poem, and its aesthetic appeal/their liking of the poem. After a blank interval of 1 sec following the last rating, the next trial started automatically with a new fixation cross. Once observers gazed at the cross, the next poem appeared.

At the end of the reading phase (which lasted about 35 minutes in total), participants were given a break of some 5 minutes (in which they stayed in the experimental room). Subsequent to this, they were informed that, in the next phase, they would be presented with haiku they had already read (72 poems) as well as new haiku they had not seen before (36 ‘foils’; participants were not told the ratio of foils to already read haiku); the task was to identify the haiku they had read (yes/no recognition response) and indicate the confidence associated with this decision. Eye-tracking was continued during this phase.

Altogether, these three phases took about 1 hour to complete.

### Data analysis

For the analysis of participants’ reading eye-movement parameters (gaze durations, fixations), we compared the effects of our experimental variables in a Bayesian ANOVA-type analysis. The Bayes Factor of a given main effect or interaction was obtained by comparing a linear model including the effect of interest (e.g., main effect of haiku type, i.e., more pronounced difference in fragment vs. phrase line reading times for juxtaposition compared to context–action haiku; cf. 1) to a null model which omits the effect (as implemented in the R package BayesFactor by Morey & Rouder: 55). Poem number (for the analysis of formal language variables; see Appendix) and participant number (for the analysis of participants’ reading times) were always included as random effects. We used the suggested default variance priors for linear models with a scale parameter of √2/2 (55). A main effect or interaction was considered ‘substantial’ if the Bayes Factor was greater than 3. A Bayes factor less than 1/3 was considered as ‘substantial’ evidence for the absence of a main effect or interaction (56). Bayes Factors in-between these thresholds would indicate that the evidence for or against an effect was ‘inconclusive’. For direct comparisons, we used two-tailed paired Bayesian t-tests. For these, we assumed a Cauchy distribution of the standardized effect sizes with the scale parameter r = √2/2 over the interval 0 to ∞, which has been suggested as a default prior for psychological research (57).

## Results

Data were analyzed using R (58), and Bayes Factors were calculated using the package BayesFactor (55). Using SR Research default settings, eye movements were classified as saccades if their speed exceeded 35°/sec and their acceleration 9500°/sec^2^. The eye movement record was stored and later on analyzed off-line using SR Research Data Viewer (version 3.1.97). The first saccade was defined as the first eye movement landing 0.8° to the right of the fixation cross. 46.23% of the trials were automatically terminated when reading time exceeded 12 sec (timed-out trials); all other trials were terminated manually, with a button press, by the participants after an average reading time of 5.52 sec. Both timed-out and manually terminated trials were included in the analyses. The results from the analysis of oculomotor variables will be presented in two sections: first analyses of overall dwell times and second, analysis of first- vs. second-/third-pass reading. In the latter, separate analyses were performed for forward- and backward-directed eye movements (progressions and regressions, respectively). For these, only fixations following progressive and regressive saccades within individual poem lines were considered.

### Analysis of overall dwell times

As can be seen from Table 1, in L.1-cut haiku, the total fixational dwell time per word is longest in the line be-fore the cut (line 1), yielding a cut effect (i.e., differential per-word dwell time between the fragment and remote phrase line) of 258 msec (fragment line 1 vs. remote phrase line 3: 1008 vs. 750 msec). In L.2-cut haiku, by contrast, dwell time is longest in the line after the cut (line 3), yielding a cut effect of 249 msec (line 3 vs. line 1: 1015 vs. 766 msec). Further, the cut effect was strong-er for juxtaposition haiku (fragment vs. phrase line: 1050 vs.733 msec; cut effect of 317 msec) relative to context–action haiku (972 vs. 783 msec; cut effect of 189 msec). For one-image (control) haiku, by contrast, the dwell times were equivalent for the two marginal (first and last) lines (line 1 vs. line 3: 709 vs. 710 msec). These obser-vations were substantiated statistically: an analysis of the cut effects per word (dwell time fragment minus dwell time [remote] phrase line) by means of a one-way ANO-VA revealed substantial evidence for the effect of poem type (juxtaposition, context–action, one-image): BF10=6.3+e7.

**Table 1:** Total dwell times (in milliseconds; number of fixations given in parentheses) per word, corrected for differential line lengths (in terms of number of words), for the three poem lines. As can be seen, readers spent overall more time in the fragment line (line 1 in L.1-cut haiku and line 3 in L.2-cut haiku) relative to the [remote] phrase line (line 3 in L.1-cut and line 1 in L.2-cut haiku). The extended time spent on the fragment line is referred to as cut effect (last row of table). Abbreviations: abs=absent; pre=present.

	**type of haiku**								
	juxtaposition				context-action				one-image
	**placement of cut**								
	L.1-cut haiku		L.2-cut haiku		L.1-cut haiku		L.2-cut haiku		N/A
	**cut marker**								
	abs	pre	abs	pre	abs	pre	abs	pre	N/A
line 1	1109 [4.14]	928 [3.63]	754 [2.80]	770 [2.84]	1074 [4.07]	920 [3.51]	733 [2.86]	751 [2.62]	709 [2.71]
line 2	759 [3.08]	798 [3.30]	732 [2.97]	781 [3.23]	796 [3.09]	674 [2.75]	707 [2.99]	667 [2.63]	752 [2.77]
line 3	733 [2.70]	675 [2.57]	960 [3.77]	1203 [4.15]	859 [3.00]	733 [2.66]	954 [3.58]	941 [3.55]	710 [2.68]
cut effect	376 [1.44]	253 [1.06]	206 [0.97]	432 [1.31]	215 [1.07]	187 [0.85]	165 [0.72]	190 [0.93]	1 [0.03]

**Figure 3.**
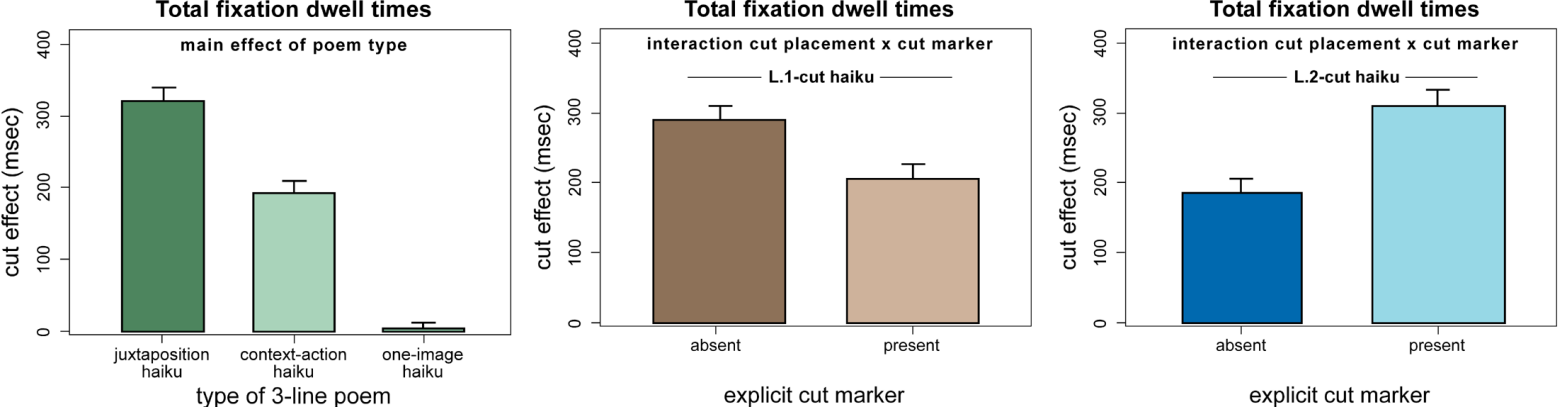
Results from the analysis of overall dwell times. Cut effects (differences in scanning time between the fragment and the [remote] phrase line) scale with semantic-conceptual features of three-line poems (juxtaposition > context-action > one-image haiku; left panel) and with their formal-structural properties, such as the use of punctuation (middle and right panels). Punctuation may render the cut between the BG and FG image perceptually/cognitively more salient and thus, in L.1-cut haiku, focus information uptake on the post-cut lines. As a result, punctuation brings about a reduction of fragment line scanning times for haiku with a cut after line 1 (L.1-cut haiku), while at the same time increasing fragment line scanning times for haiku with a cut after line 2 (L.2-cut haiku).

This suggests that the extended processing of the (frag-ment) image before the cut (L.1-cut haiku) or, respective-ly, after the cut (L.2-cut haiku) is a unique feature of two-image haiku, and not evident with one-image haiku. Moreover, the semantic distance between the two images had a major influence on reading behavior: the cut effect was more marked for juxtaposition than context–action haiku (BF10=60.41; see also Fig. 3, left panel). Of note, the overall cut effect in two-image haiku emerged early on during our (naïve) readers’ exposure to this genre of poetry and stayed relatively stable across the whole read-ing phase. Examining the cut effect (collapsed across all experimental conditions) across eight reading ‘epochs’ (of 9 haiku each) in a single-factor ANOVA failed to reveal a significant effect of epoch (in fact, the associated BF10=0.03 provides strong evidence for a null-effect). A less fine-grained t-test comparing the cut effect between the first and second half of the reading phase (239 vs. 267 msec) also turned out non-significant (BF10=.28). Thus, the fact that the cut effect was at most only slight-ly (numerically) increased in the second half of the read-ing phase makes it likely that this effect is an inherent, rather an acquired, feature of the reading of two-image haiku. – This effect pattern essentially corroborates our previous findings (1,30).

New findings relate to the effects arising from the presence (vs. absence) of explicit punctuation to emphasize the cut in two-image haiku (see also the middle and right panels of Figure 3). These cut marker effects proved strongly dependent on the placement of the cut at the end of line 1 vs. the end of line 2 as revealed by a substantial cut location × cut marker interaction (BF10 = 4.89; ANOVA of cut effects with the factors *poem type*: juxtaposition vs. context–action; *cut location*: L.1- vs. L.2-cut haiku; and cut marker: present vs. absent). For L.1-cut haiku (with a cut at the end of line 1), the cut effect was reduced, by 76 msec, when the poem contained an explicit cut marker (cut marker present vs. absent, 220 vs. 296 msec; BF10=2.83). The opposite was true for L.2-cut haiku (with a cut at the end of line 2): the cut effect was 125 msec stronger when the haiku contained an explicit marker (marker absent vs. present: 311 vs. 186 msec; BF10=8.63). This differential pattern suggests that encountering a cut marker at the end of the fragment line (in L.1-cut haiku) renders the (phrase) lines following the cut cognitively more salient, directing information uptake towards these lines and thus reducing the cut effect for L.1-cut haiku. For analogous reasons, explicit cut markers would enhance the cut effect for L.2-cut haiku, as meaning resolution would require increased information uptake in the post-cut fragment line (line 3).

### Differential cut-marker dynamics between L.1- and L.2-cut haiku

As the cut effect (and its modulation by an explicit marker) is reflected in a difference score – the differential reading time between the fragment and phrase lines (technically in the analyses above: the remote phrase line) – , it is interesting to examine more closely how each of these lines contributes to the effect pattern.

*L.1-cut haiku*: Examining the total dwell times (per word) accumulated over all three poem lines reveals that, for L.1-cut haiku, the reading time is overall reduced when there is an explicit cut marker vs. when there is not: 2,364 vs. 2,665 msec, BF10=507.96; that is, the presence of a cut marker yields total savings of 301 msec per word. Of these total savings, 167 msec (55%) originate from the single fragment line, as compared to 42 and 92 msec from the first and the second/remote phrase line, respectively. Thus, the presence of a cut marker benefits mainly the ‘working-out’ of the fragment line, though the phrase lines benefit as well (albeit to a lesser extent).

*L.2-cut haiku*: In contrast to L.1-cut haiku (in which an explicit cut marker gives rise to savings in reading time), for L.2-cut haiku, the total reading time is increased when there is an explicit cut marker vs. when there is not: 2,556 vs. 2,421 msec, BF10=12.42, i.e., the cut maker yields total costs of 135 msec. Of these, 115 msec (85%) are attributable to the fragment line (as compared to only 4 and 15 msec to the first/remote and the second phrase line, respectively).

### Differential cut-marker dynamics between ellipsis and dash markers

Given these cut marker effects, we went on to ask whether the marker effects would differ depending on the type of marker used to emphasize the cut in the present poem sample: ellipsis vs. dash markers. Note that this is a post-hoc analysis we could perform only for juxtaposition haiku, because we had an equal number of ellipsis and dash markers in the two cut-position conditions (L.1-cut, L.2.-cut) only for this type of poem (for context–action haiku, poems with an ellipsis marker were rare, so that any marker-type effects obtained might really be owing to properties of these particular haiku). This analysis did reveal ellipsis markers to function differently, in terms of eye-movement patterns and cut effects, to dash markers.

In more detail, for comparing the effects of the two types of cut markers, we computed (1) the cut effect and (2) the modulation of the cut effect by the presence of an explicit marker for each experimental condition (cut position: L.1-cut, L.2-cut; cut marker: ellipsis, dash). For assessing the modulatory effect of the cut markers, we subtracted cut effects in the respective (ellipsis, dash) marker condition from those in the marker-absent baseline. Differences in the impact of individual cut markers (ellipsis, dash) on reading should be revealed by systematic variations of this *cut-effect difference measure*.

For L.1-cut haiku, the cut effect was overall (i.e., in terms of the overall reading times per word) comparable between the dash and the (marker-absent) baseline conditions (366 vs. 376 msec; BF10=.23, cut-effect difference: –10 ms, with the negative sign indicating a reduction of the cut-marker effect), whereas it was significantly reduced with ellipsis markers (243 vs. 376 ms; BF10=8.12, cut-effect difference: –133 ms). In contrast, for L.2-cut haiku (in which the presence of a marker generally increased the time taken to read the fragment line, thus increasing the cut effect), dash markers increased the overall reading time by 310 msec relative to the baseline (cut effects of 505 vs. 195 msec, BF10=23.54), which compares to an increase of 165 msec (i.e., effectively half the size) for ellipsis markers (360 vs. 195 msec; BF10=6.28). These observations were substantiated by a cut placement × marker type interaction, BF10=12.73.

Thus, taken together, the evidence suggests that the specific cut markers are being noticed and induce differential reading patterns, dependent on the cut position, with ellipsis markers being more facilitative (cut at end of line 1) or less interruptive (cut at end of line 2) than dash markers.

### Analysis of first- and second-/third-pass reading

While the analysis of the overall dwell times showed that the difference in (per-word) dwell times between the fragment and phrase lines varies as a function of both semantic-conceptual (semantic distance between images) and formal-structural (cut marker/punctuation) features of the two-image haiku, it remains unclear when, during the reading of these haiku, these differences actually arise. When examined at such a more ‘on-line’ level, we (1) found the reading of haiku to involve highly non-linear patterns of eye movements: readers go forwards and backwards within lines (with a greatly increased rate of regressions within lines compared to that reported by, e.g., 59, for normal text reading), and they jump between lines not only in the standard, forward path, but they also go back, for instance, from the end to the beginning of the poem. Thus, frequently, a poem is sampled not only once, but two or three times. To deal with this complexity and gain a more detailed picture of the re-/reading dynamics in the present study, we went on to examine the sampling of two-image haiku in terms of the first-, second-, and third-pass reading of each line, taking into account differentially both progressive and regressive saccadic movements (see, e.g., 60 for a similar analytical approach to examining eye movements in the global reading comprehension of longer texts). These passes accounted for about 85% of the overall reading times (re-readings beyond a third-pass, accounting for some 15% of reading activity, occurred too rarely to permit meaningful analysis). Although the vast majority of eye movements progresses with the text, readers may not necessarily fixate each word or re-read a word already during the initial, first-pass scan. In (non-literary) text reading, approximately 25% of saccades move the eyes in the direction opposite to reading direction (61). Of note, this percentage appears to be doubled in the reading of ELH (cf. 1, 30) or poetry in general (35, 36). Accordingly, we analyzed the dwell times following progressive (left-to-right) and regressive (right-to-left) saccades within lines – henceforth referred to as ‘progressive’ and, respectively, ‘regressive dwell times’ – separately for the various conditions of two-image haiku.

It is relatively undisputed that first-pass reading times indicate processes associated with the initial interpretation of a text region (line), while second- (and higher-)pass reading reflects the re-evaluation of the initial interpretation (62). In addition, there is robust evidence that discourse incongruence effects – and thus the type of effects that also underlies the ‘cut’ in haiku – already become manifest during first-pass reading (e.g., 63, 64).

Technically, first-, second-, and third-pass reading times were obtained by summing fixation durations (and numbers) following progressive and regressive saccades within (rather than across) individual poem lines. When the eyes left a certain line – either forward by entering a subsequent line or backward by entering a previous line – the respective (first-, second-, third-)reading pass was considered to be complete for that line. Since both progressive and regressive eye movements decreased with increasing reading passes (first- vs. second- vs. third-pass: 40% vs. 27% vs. 18% of overall reading times), reading times were collapsed across the second and third passes to obtain a reasonably stable picture of the ‘late’ phases of reading (henceforth referred to as ‘second-/third-pass’ reading). In terms of statistical analysis, for both progressive and regressive dwell times, we first determined the cut effect (fragment line reading times minus [remote] phrase line dwell times) and examined this effect as a function of our three experimental variables: haiku type, cut placement, and cut marker, as well as comparing the cut effects between the first and the second reading pass. Tables 2 and 3 summarize the data separately for fixational dwell time activity following progressive and, respectively, regressive saccades.

There are four main findings. First, the ratio of (within-line) progressive to regressive saccades is 1:0.74 overall. In other words, some 40% of saccades are regressions: approximately 16% in line 1, 11% in line 2, and 15% in line 3. While these line-specific ratios differ little as a function of the experimental variables (haiku type, placement of cut, and explicit cut marker), they differ greatly between first- and second-pass reading: more reading time was spent in individual poem lines following regressive saccades in the second reading pass (in which the ratio between progressive and regressive fixations was balanced: 1.00:1.02, i.e., ca. 50% of saccades were regressions) as compared to the first pass (in which the ratio was 1.00:0.46, i.e., ca. 30% of saccades were regressions). An ANOVA of pro- vs. regressive fixation activity in the first vs. the second pass yielded substantial evidence for the interaction [BF10=1.94e+09]. In other words, while two-image haiku are sampled relatively linearly, in a predominantly forward-directed scan, during first-pass reading, re-reading is more disfluent, involving increased backward-directed scanning during the second pass. Interestingly, an almost identical pattern was observed with one-image haiku (first pass: 1.00:0.50, i.e., 33% regressions; second pass: 1.00:0.94, i.e., 49% regressions; interaction fixation type × reading pass: BF10=91.38). This indicates that the pass-dependent increase in regressive eye movements is not an exclusive feature of two-image haiku.

**Figure 4.**
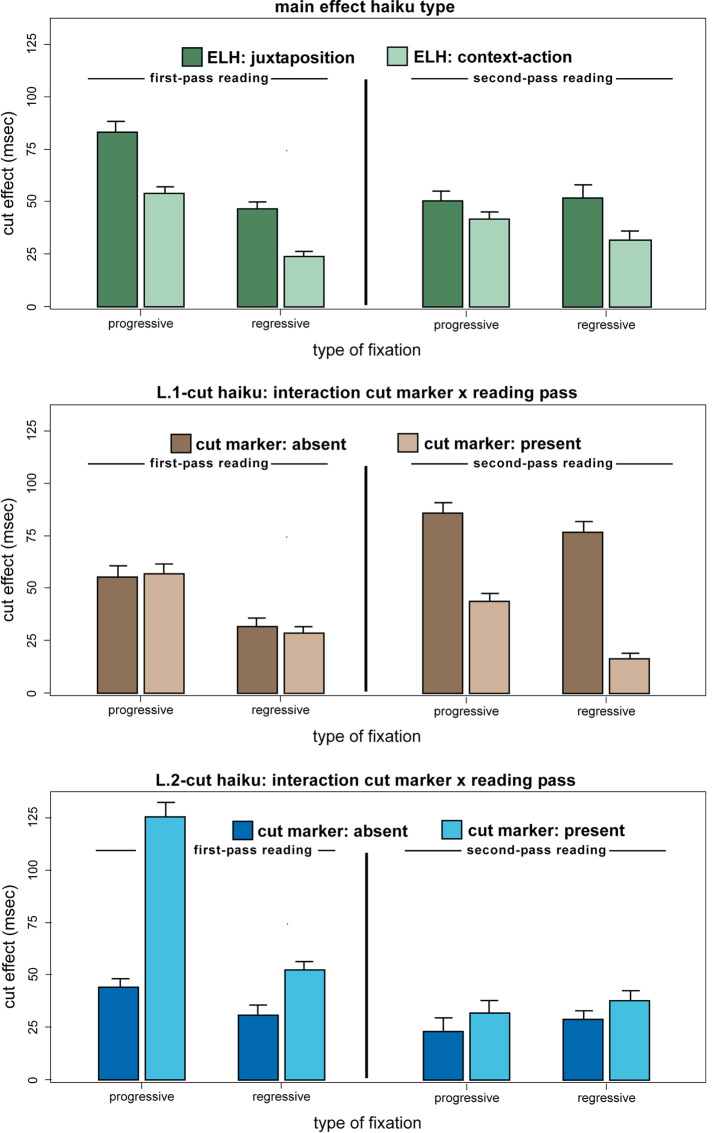
Cut effects (fragment line minus [remote] phrase line reading times, in milliseconds) as a function of progressive vs. regressive fixations in first- vs. second-pass reading. The three panels show re-/reading differences arising from different semantic-conceptual ELH properties (juxtaposition vs. context–action haiku; top panel) and different formal-structural ELH properties (non-/use of cut markers in L.1- vs. L.2-cut haiku; middle and bottom panel).

Second, dwell times were overall longer in the fragment compared to the [remote] phrase line, with this pattern being particularly pronounced for juxtaposition haiku. While this mirrors the pattern manifest in the overall reading times (see analysis above), a breakdown of the data into distinct reading phases revealed that an elevated cut effect for juxtaposition vs. context–action haiku was manifest already during first-pass reading, following both progressive saccades (juxtaposition vs. context–action haiku: 82 vs. 55 msec; BF10=4.28) and regressive saccades (45 vs. 24 msec; BF10=153.30). This pattern persisted during second-/third-pass reading, again following both progressive (50 vs. 39 msec; BF10=2.87) and regressive saccades (51 vs. 29 msec; BF10=18.23). This differential cut effect was substantiated by a haiku type main effect (BF10=18.90; ANOVA of the cut effect with the factors haiku type: juxtaposition vs. context–action; saccade direction: progressive vs. regressive; reading pass: first- vs. second-/third-pass; see top panel of Fig. 4).

Third, the effect of the presence vs. absence of an explicit cut marker was more pronounced in later reading passes, compared to the first pass – but, critically, only for L.1-cut haiku (interaction cut marker × reading pass: BF10=6.46; ANOVA of the cut effect with the factors cut position: L.1- vs. L.2-cut haiku; *saccade direction*: progressive vs. regressive; *reading pass*: first- vs. second-/third-pass; see middle panel of Fig. 4). In first-pass reading of L.1-cut haiku, the cut effects were comparable between conditions with and without punctuation (cut marker present vs. absent: progressive saccades, 55 vs. 54 msec, BF10=.23; regressive saccades, 27 vs. 30 msec, BF10=.23); in second-pass reading, by contrast, statistically *less* time was spent in the reading of the fragment line 1 when a cut marker was present at the end of this line (cut marker present vs. absent: progressive fixations, 40 vs. 84 msec; BF10=1.06+e3; regressive fixations, 17 vs. 76 msec; BF10=2.98+e4). This pattern looks as if, with L.1-cut haiku (of whatever type), readers initially disregard the explicit cut marker and take in the poem in a relatively linear sweep, across the fragment and phrase lines, in first-pass reading – rather than dwelling extendedly on the fragment line. However, as also depicted in Figure 4, fragment line re-/reading dwell times show a saving during the second pass in the presence (vs. the absence) of a cut marker. This suggests that the cut marker is in fact noted during first-pass reading and, compared to the absence of an explicit marker, comes to expedite the integration of the poem’s two images into a coherent meaning in second-pass reading.

Fourth, as can also be seen from Figure 4, in L.2-cut haiku, the cut effect was particularly marked when readers did (vs. did not) encounter a cut marker at the end of the phrase (in line 2). However, this effect was seen only during first-pass reading for progressive dwell times (124 vs. 40 msec, BF10=203.63) and (with anecdotal evidence) for regressive dwell times (52 vs. 30 msec, BF10=1.87; cut marker present vs. absent, respectively). During second-pass reading, by contrast, the (increased) dwell times in the fragment line were comparable between conditions with and without a cut marker (progressive fixations, 31 vs. 23 msec, BF10=.25; regressive fixations; 40 vs. 28 msec, BF10=.30). This effect pattern was substantiated by a cut marker × reading pass interaction [BF10=4.90; ANOVA of the cut effect with the factors *cut position*: L.1- vs. L.2-cut haiku; *saccade direction*: progressive vs. regressive; *reading pass*: first- vs. second-/third-pass; see bottom panel of Fig. 4]. Thus, the key finding is that with L.2-cut haiku, punctuation effects became manifest already during first-pass reading. This would be in line with the suggestion (made above with reference to L.1-cut haiku) that encountering a cut marker immediately prompts meaning resolution processes in the post-cut lines, which influences the processing of the fragment line (line 3) in L.2-cut haiku – thus enhancing the cut effect already in first-pass reading.

However, the following finding is seemingly inconsistent with this interpretation: In L.1-cut haiku, the presence of a cut marker produces ‘savings’ in total reading time (i.e., assuming that participants followed the instruction to try to understand the haiku’s meaning, meaning resolution was achieved faster when a cut marker was present vs. absent). In L.2-cut haiku, by contrast, the marker gives rise to an overall ‘cost’.

**Table 2:** Fixational dwell times (per word) following progressive saccades during first-pass (top-half) and second-pass (bottom-half) reading in milliseconds (number of fixations given in parentheses). Dwell times are normalized per word to correct for differential line lengths in terms of word number among the three poem lines. The extended time spent on the fragment line (line 1 in L.1-cut and line 3 in L.2-cut haiku) relative to the [remote] phrase line (line 3 in L.1-cut and line 1 in L.2-cut haiku) represents the cut effect.

**Left-to-right reading (progressions)**									
	**type of haiku**								
	juxtaposition				context-action				one-image
	**placement of cut**								
	L.1-cut haiku		L.2-cut haiku		L.1-cut haiku		L.2-cut haiku		N/A
	**cut marker**								
	abs	pre	abs	pre	abs	pre	abs	pre	N/A
first-pass reading									
line 1	280 [1.09]	266 [0.88]	216 [0.87]	188 [0.66]	251 [0.94]	243 [1.02]	218 [0.81]	192 [0.74]	193 [0.72]
line 2	170 [0.60]	185 [0.62]	166 [0.71]	164 [0.58]	151 [0.60]	142 [0.53]	167 [0.68]	158 [0.56]	143 [0.61]
line 3	204 [0.86]	189 [0.78]	246 [0.89]	332 [1.22]	220 [0.88]	210 [0.93]	269 [0.95]	295 [1.00]	196 [0.73]
cut effect	77 [0.23]	77 [0.11]	29 [0.02]	144 [0.57]	31 [0.06]	33 [0.09]	52 [0.14]	103 [0.26]	3 [0.01]
second-/third-pass reading									
line 1	253 [0.92]	197 [0.73]	157 [0.69]	170 [0.69]	266 [1.11]	191 [0.69]	160 [0.53]	152 [0.56]	146 [0.60]
line 2	130 [0.51]	133 [0.53]	130 [0.55]	126 [0.49]	138 [0.61]	120 [0.49]	142 [0.49]	112 [0.41]	127 [0.49]
line 3	163 [0.55]	140 [0.46]	182 [0.66]	198 [0.79]	188 [0.73]	167 [0.65]	182 [0.66]	185 [0.78]	149 [0.64]
cut effect	91 [0.37]	57 [0.27]	25 [-0.03]	28 [0.10]	78 [0.37]	23 [0.03]	22 [0.13]	33 [0.22]	3 [0.04]

**Table 3:** Fixational dwell times (per word) following regressive saccades during first-pass (top-half) and second-pass (bottom-half) reading in milliseconds (number of fixations given in parentheses). Dwell times are normalized per word to correct for differential line lengths in terms of word number among the three poem lines. The extended time spent on the fragment line (line 1 in L.1-cut and line 3 in L.2-cut haiku) relative to the [remote] phrase line (line 3 in L.1-cut and line 1 in L.2-cut haiku) represents the cut effect.

**Right-to-left reading (regressions)**									
	**type of haiku**								
	juxtaposition				context-action				one-image
	**placement of cut**								
	L.1-cut haiku		L.2-cut haiku		L.1-cut haiku		L.2-cut haiku		N/A
	**cut marker**								
	abs	pre	abs	pre	abs	pre	abs	pre	N/A
first-pass reading									
line 1	139 [0.51]	131 [0.58]	108 [0.39]	98 [0.34]	125 [0.46]	123 [0.44]	110 [0.42]	102 [0.40]	95 [0.37]
line 2	85 [0.34]	94 [0.42]	83 [0.30]	82 [0.36]	75 [0.27]	72 [0.30]	85 [0.36]	77 [0.34]	72 [0.25]
line 3	97 [0.42]	92 [0.31]	140 [0.57]	165 [0.55]	108 [0.38]	108 [0.40]	138 [0.49]	138 [0.56]	99 [0.39]
cut effect	42 [0.09]	39 [0.27]	32 [0.18]	67 [0.21	18 [0.09]	15 [0.04]	28 [0.07]	36 [0.17]	4 [0.02]
second-/third-pass reading									
line 1	241 [0.92]	184 [0.75]	149 [0.59]	168 [0.69]	233 [0.81]	163 [0.59]	156 [0.57]	154 [0.53]	135 [0.52]
line 2	130 [0.56]	130 [0.46]	128 [0.43]	120 [0.47]	132 [0.47]	117 [0.43]	138 [0.59]	107 [0.46]	123 [0.42]
line 3	154 [0.53]	149 [0.55]	179 [0.71]	222 [0.77]	167 [0.57]	164 [0.67]	182 [0.70]	180 [0.73]	141 [0.49]
cut effect	87 [0.39]	35 [0.20]	30 [0.12]	54 [0.08]	66 [0.24]	-1 [-0.08]	25 [0.12]	26 [0.20]	6 [0.03]

### Differential cut-marker dynamics between L.1- and L.2-cut haiku

*L.1-cut haiku*: For L.1-cut haiku, the above analysis of the total reading times (per word) disclosed substantial savings when a cut marker was present (vs. absent), with the savings originating mainly in the fragment line. A more detailed, pass-based analysis of this effect revealed that, while there were some numerical savings already during first reading (almost entirely due to savings on progressive dwells: 618 vs. 638 msec, BF10=.37), there were substantial savings in the second/third pass, in both progressive (474 vs. 569 msec; savings=95 ms, BF10=91.39) and regressive fixations (453 vs. 528 msec; savings=75 ms, BF10=24.38), again largely due to shortened dwell times in the fragment line (savings on progressive and regressive dwells: 66 msec 70% and 63 msec 85%, respectively). Thus, in addition to fostering a somewhat more linear scan of the poem in the first pass, the cut marker produces a particularly marked benefit on the return (on subsequent passes) to the fragment line, where both progressive and regressive dwells are reduced, indicative of a swifter re-appraisal of the fragment image. This then also benefits the further (re-)reading of the phrase lines, suggestive of an expedited meaning wrap-up. Thus, overall, the marker renders reading more fluent, facilitating meaning construction/integration (if only by making readers more aware of what the main challenge is for achieving understanding).

*L.2-cut haiku:* In contrast to L.1-cut haiku, for L.2-cut haiku, the presence (vs. absence) of a cut marker yielded costs in the total reading times, attributable mainly to the fragment line (see analysis of total reading times above). These costs were largely generated in the first pass, and exclusively so by extended progressive dwells in the fragment line (–56 msec, BF10=28.99). This pattern re-occurred, in a much shallower form, in second-/third-pass reading, with fragment line costs of –10 msec (BF10=.35) and –21 msec (BF10=.99) on progressive and regressive fixations, respectively. Thus, immediately upon encountering a cut marker at the end of line 2, reading slows down markedly on the forward path in the fragment line, though without increased regressive (‘re-checking’) activity, perhaps indicative of the reader being taken by surprise/being puzzled. Increased regressive re-checking is deferred to re-reading the fragment line, indicative perhaps of an effort to work out how the fragment bears on the phrase. This pattern suggests that, in contrast to L.1-cut haiku, the marker heightens the element of surprise evoked by the cut (which slows processing in the fragment line) in the first pass, and then induces some recovery process over the subsequent passes.

### Differential cut-marker dynamics between ellipsis and dash markers

For L.1-cut (juxtaposition) haiku, analysis of the total reading times had shown that the cut effect was overall comparable between the dash and the (marker-absent) baseline, whereas it was significantly reduced relative to the baseline with ellipsis markers. As revealed by a pass-based analysis, this reduction arose largely (with anecdotal evidence) in the second/third reading pass (cut-effect difference, first vs. second pass: –33 msec vs. –94 msec, BF10=1.81) for ellipsis markers, which compares with +21 vs. –30 msec for dash markers, BF10=1.72). For L.2-cut haiku, by contrast, the differential increase in the overall cut effect between dash and ellipsis markers (both compared to the baseline) arose mainly in the first reading pass (cut-effect differences: +179 vs. +83 msec for dash vs. ellipsis markers; BF10=3.99), though with a substantial contribution also in the second/third pass (+107 vs. +46 msec, BF10=2.52). Thus, ellipsis markers (as compared to dash markers) facilitate mainly the re-reading of L.1-cut haiku, while they are less interruptive to the initial reading of L.2-cut haiku.

### Analysis of subjective ratings

Reading of each individual haiku was followed by a set of rating questions probing: understanding achieved, feelings of surprise, sudden insight (‘aha’ experience), emotional valence (joyful vs. sad), emotional arousal, aesthetic appeal, and liking. Table 4 summarizes the effects of our experimental manipulations for each rating. As can be seen, any differences between the rating scores were subtle, typically only a fraction of a rating scale unit. For statistical analysis, we computed a 2 (haiku type: juxtaposition vs. context-action) × 2 (cut placement: L.1-cut vs. L.2-cut haiku) × 2 (cut marker: present vs. absent) repeated-measures (Bayesian) ANOVA for each subjective measure. The results revealed main effects of cut position and haiku type for the two measures ‘understanding’ and ‘insight’ (both BF10 > 7.8): scores were overall higher for L.1- than for L.2-cut haiku (understanding: 3.07 vs. 2.81; insight: 2.20 vs. 1.94) and higher for context-action than for juxtaposition haiku (understanding: 3.20 vs. 2.69; insight: 2.19 vs. 1.95). As regards emotion arousal, the main effect of cut position was significant (BF10= 4.64): arousal as greater for haiku with a cut at the end of line 1 than with a cut at the end of line 2 (2.31 vs. 2.09). The only other significant effect was revealed for ‘emotional valence’, namely, a haiku type × cut position × cut marker interaction (BF10=8.07+e5): for juxtaposition haiku, rated valence was somewhat more negative for L.2- than for L.1-cut haiku when a cut marker was present (1.97 vs. 2.41), but more positive when a cut marker was absent (2.27 vs. 1.90); for context-action haiku, by contrast, rated valence was generally more positive for L.2- than for L.1-cut haiku, with (2.30 vs. 2.03) or without a cut marker (2.38 vs. 2.15). Note that the various (haiku type x cut position x cut marker) conditions were not (a-priori) equated in terms of emotional valence (in contrast to a host of linguistic variables; see Appendix), so that this interaction may simply reflect an uncontrolled bias towards ‘sad’ emotional valence in one set of the sample poems (context-action x L.2-cut x marker-present condition). – No significant effects (neither main effects not interactions: all BF10 < 1) were found for ratings of ‘surprise’, ‘liking’ (if anything, L.2-cut juxtaposition haiku were liked least: 2.33 vs. 2.57 combined across the other three cut position x haiku type conditions), and ‘emotional arousal’.

**Table 4:** Mean subjective ratings on all seven rating scales (understanding achieved, feelings of surprise, ‘aha’ experience, joyful vs. sad emotional valence, emotional arousal, aesthetic appeal, and liking) as a function of our (haiku type, placement of cut, cut marker) experimental manipulations. The right column shows the ratings obtained for one-image (control) haiku.

**Subjective ratings**									
	**type of haiku**								
	juxtaposition				context-action				one-image
	**placement of cut**								
	L.1-cut haiku		L.2-cut haiku		L.1-cut haiku		L.2-cut haiku		N/A
	**cut marker**								
	abs	pre	abs	pre	abs	pre	abs	pre	N/A
Understanding achieved	2.89	2.84	2.66	2.35	3.34	3.22	3.19	3.03	3.39
Surprise	2.12	1.91	1.72	1.94	2.07	2.04	1.92	1.90	1.94
Insight (‘aha’)	2.14	2.02	1.83	1.80	2.32	2.30	2.13	2.00	2.28
Emotional valence (1=sad, 4:=joyful)	1.90	2.41	2.27	1.97	2.15	2.03	2.38	2.30	2.15
Emotional arousal	2.29	2.19	2.04	2.00	2.38	2.40	2.13	2.20	2.37
Aesthetic appeal	2.57	2.82	2.51	2.54	2.57	2.61	2.71	2.67	2.76
Liking	2.50	2.56	2.31	2.35	2.64	2.56	2.65	2.53	2.73

Further, comparisons (by means of direct t tests) between on-image (control) haiku and two-image haiku combined revealed ‘understanding achieved’ and, less conclusively, ‘sudden insight’ to be higher for one-image haiku (understanding: 3.39 vs. 2.94. BF10 = 4.7+e5; insight: 2.28 vs. 2.07, BF10 = 1.89). A similar pattern was found for ‘liking’ (2.73 vs. 2.51, BF10 = 3.01) and, less conclusively, ‘emotional arousal’ (2.37 vs. 2.20, BF10 = 1.98). There were no differences in terms of ‘surprise’ (1.94 vs. 1.95, BF10 = .23), ‘emotional valence’ (2.15 vs. 2.17, BF10 =. 24), and ‘aesthetic appeal’ (2.57 vs. 2.65, BF10 = .63).

## General Discussion

### Genre-specific semantic and structural properties modulate the reading of ELH

The aim of the present study was to examine the patterns of eye movements during the reading of normative, three-line ELH with a clearly discernible cut between the fragment and phrase images. In these haiku, the break between the – on first encounter, often seemingly discrepant – images attracts attention, making readers adopt a more disfluent, ‘controlled’ reading mode in an effort to bridge the gap and achieve meaning resolution. Structurally, the cut is positioned either at the end of line 1 (fragment in line 1) or at the end of line 2 (fragment in line 3), and it can be marked/emphasized by punctuation. These structural properties are orthogonal to the type of haiku, context–action vs. juxtaposition, which differ in the degree of semantic discrepancy between their two component images, the fragment image and the phrase image. Our aim was to track the influence of these formal-structural and semantic-conceptual features typical of ELH as a genre in readers’ eye-movement behavior. By also including a condition of one-image, ‘uncut’ haiku, we aimed to delineate the pattern of cut effects (as expressed in the oculomotor measures for ‘cut’, two-image haiku) against the processing of these ‘uncut’ haiku which were expected to give rise to a more fluent mode of reading throughout. – Our main findings, and their implications, are summarized and discussed below.

(1) **The position of the cut in two-image haiku was confirmed to have a major, and general, influence on the eye-movement pattern**: Overall more reading time per word was spent in one particular line, the fragment (line 1 in L.1-cut ELH and line 3 in L.2-cut ELH), as compared to (each of) the phrase lines. This general ‘cut effect’ occurred independently of the type of two-image haiku (context–action or juxtaposition), the position of the cut (at the end of the first or the second line), and the presence versus absence of a cut marker. Importantly, no comparable effect was found with one-image haiku: the reading patterns for these poems do not show a concentration of scanning activity on any particular line.

Thus, from the pattern of overall reading times alone, we can tell, or even ‘predict’, whether and where there is a cut in a three-line haiku. The extended time readers spent processing the fragment is highly likely due to them encountering the cut. Within the theoretical framework of (Neuro-)Cognitive Poetics, this can be taken to indicate that the cut acts as a foregrounding, attention-invoking feature, putting the reader into a relatively disfluent, ‘controlled’ reading mode, characterized by increased (progressive and regressive) eye-movement activity within the fragment line. That is, the reader treats the fragment as being pivotal for global meaning construction: it is, ultimately, in the fragment line that the tension between the juxtaposed images is resolved.

On a more basic level, the systematic occurrence of the cut effect as such can be seen as an indicator that our readers indeed worked towards constructing a coherent situation model for the poems: evidence from reading proficiency research indicates that inconsistency detection and inconsistency resolution presuppose the construction of a global situation model for the text (e.g., 65).

(2) **Both the formal-structural variable of the placement of the cut and the semantic-conceptual variable of haiku type modulate the basic cut effect differentially**. While being evident in the overall reading times, the effects of these variables emerge in characteristic ways over the course of the initial sampling (first-pass) and subsequent (second- and third-pass) re-reading of the haiku.

Concerning the haiku’s semantic-conceptual features, the (total) cut effect was more marked for juxtaposition than for context–action haiku, independently of whether the cut occurred at the end of line 1 or at the end of line 2. In other words, the cut effect reflects the strength of the semantic-conceptual distance, or discrepancy, between the two image components, which is generally greater for juxtaposition than for context–action haiku: the larger the gap, the more (progressive and regressive) eye-movement activity is focused on the fragment image. Importantly, this modulation arose already during first-pass reading, and continued when re-entering the fragment line in subsequent passes. This indicates that, with both types of haiku, meaning construction starts already during the first pass (indicated by extended dwell times in the fragment over the phrase lines) and is refined during subsequent reading passes. These core findings are in line with reports of differential early and late incongruence effects in the reading of other text types (e.g., 63, 64).

What our data demonstrate in addition is that the degree of discourse-semantic incoherence, which is operationalized here via the different haiku types, has a systematic impact on eye-movement patterns. In juxtaposition haiku, the poem’s fragment image – even though it may be relatively non-ambiguous in itself (e.g., “bruised apples” in Melissa Allen’s poem, see Fig. 1) – would typically be more semantically remote from the phrase image (i.e., more ‘indeterminate’), compared to the more situational, ‘context’ image in the fragment of context–action haiku. Accordingly, the increased activity (progressions and regressions) in the fragment line may reflect the increased difficulty/effort of constructing the ‘bridging context’ determining the fragment’s meaning in relation to the phrase. And the amount of time required to elaborate and settle on a fitting interpretation would depend on when the fragment image is encountered: before or after the phrase image. If encountered after the phrase, working out a possible relationship would already be informed, or ‘constrained’, by the prior reading of the phrase lines, and the fit of any emerging (potentially competing) interpretation(s) could be assessed directly in the fragment line. Thus, in L.2-cut haiku, both the elaboration of plausible relationships and the assessment of their fit would be concentrated on the fragment line, giving rise to a large cut effect. By contrast, if the fragment is encountered before the phrase, while some, ‘salient’ interpretation(s) may immediately be evoked in the fragment line, the matching process (elaboration and assessment of fit) would have to be deferred to the subsequent reading of the phrase lines, thus reducing the cut effect in L.1-cut haiku.

While this pattern would be similar for context–action haiku, with this type of poem, less mental effort would be required to align the two images because the situation model and its fit with the action taking place within this context is easier to determine – not least also because the context–action relation – as an instantiation of the basic figure–ground schema (24, 66, 67) – is perhaps one of the most fundamental schemas available to us to construct ‘episodic’ representations in the first place.

Also, this proposal – of two processes: elaboration of relationships and assessment of fit – could account for the absence of (marked) cut effects in subsequent reading passes: the latter may serve to confirm some already favored solution, and readers would engage in an extended rechecking mode (which would be reflected in further cut effects) only if the preferred solution is dismissed on second reading.

(3) Concerning the more formal-structural haiku features, **the effect of cut position (extended time spent on the fragment line) was modulated by the presence of explicit punctuation (cut markers), irrespective of haiku type**. Encountering the marker led to prolonged ‘dwelling’ on the line/s immediately after the cut, that is: lines 2 and 3 in L.1-cut haiku and line 3 in L.2-cut haiku. As a result, the cut effect was reduced for L.1-cut haiku, because more time was spent overall in the post-cut phrase line/s (cut effects, in terms of total dwell time, of 220 vs. 296 msec when a marker was present vs. absent). For analogous reasons, the cut effect was increased for L.2-cut haiku, because extended time was spent in the post-cut fragment line (cut effects of 311 vs. 189 msec). Overall, this pattern indicates that encountering an explicit cut marker – in the first instance: a surface-level structural feature – significantly modulates the extraction of meaning. When encountering a marker at the end of line 1, the reader might be prompted to attempt an integrative analysis of the haiku as a whole (working out and aligning the meaning of the fragment image with the phrase image) in the phrase line/s. Conversely, when encountering a marker after line 2, these processes (of working out the impact of the fragment on the already sampled phrase) are concentrated on the fragment line.

(4) The suggestion of **marker-dependent differences in meaning resolution for L.1- vs. L.2-cut haiku** is further bolstered by an analysis of first- vs. second/third-pass dwell times. In L.1-cut haiku, a visible cut marker tended to expedite the first scan of the poem (reflected in a reduced rate of regressions) and subsequently shortened the re-reading time specifically of the fragment line (expressed in both reduced progressive and regressive eye-movement activity); since re-reading of the phrase lines was relatively unaffected, the savings on the fragment line increased the cut effect in the second reading pass. With L.2-cut haiku, by contrast, the marker encountered at the end of line 2 led to an immediate slow-down in the following fragment line, as reflected by prolonged fixations following progressive saccades (rather than an increase in regressive activity). While the (subsequent) re-reading of the phrase lines differed little from the marker-absent condition, re-reading of the final fragment line exhibited a recurring, though compared to the first pass shallower, marker effect (characterized by both increased progressive and regressive activity). Thus, while the marker produced time savings (originating mainly in the second pass) with L.1-cut haiku, it gave rise to overall costs (originating mainly in the first pass, but to a noticeable extent also in the second pass) with L.2-cut haiku.

These opposing patterns may be taken to indicate that encountering a cut marked by punctuation has a disorienting effect in L.2-cut haiku, whereas the marker is actively utilized in L.1-cut haiku. In L.2-cut haiku, assuming that the phrase lines (1 and 2) of the poem are processed in a relatively fluent, forward-gliding (BG) mode, encountering the marker at the end of line 2 gives rise to surprise. This, in turn, slows down information uptake in the fragment line, without involving re-checking – perhaps indicative of the reader being startled at first and/or pausing to switch to a more attentive (FG) mode of reading. Increased re-checking (characterizing processing in FG mode) sets in in the second pass, commencing with a re-appraisal of the phrase in the light of the fragment (sampled at the end of the first pass) before proceeding to final checking and meaning wrap-up in the fragment line. On this rendering, the cost in processing time for L.2-cut haiku with (vs. without) a cut marker arises mainly in the first pass and reflects a surprise response upon encountering the punctuation. This is consistent with participants’ subjective rating of the ‘surprise’ they associated with the haiku, which is increased for haiku with vs. without a cut marker (ratings, on a four-point scale, of 1.92 vs. 1.82, BF10=2.83). Also consistent with this interpretation, the additional time taken to read L.2-cut haiku with vs. without a cut marker does not translate into a benefit in terms of the (subjective) understanding that participants feel they have achieved (in fact, there appears to be a cost: ratings, on a four-point scale, of 2.69 vs. 2.93, BF10=10.50). It would need to be seen whether disruption is something naïve haiku readers, like those who participated in the present study, show, but not readers experienced with the genre.

With L.1-cut haiku, by contrast, readers encounter the cut marker already in FG mode (evoked by the fragment in the first line), which then drives a relatively swift taking-in of the phrase, followed by a facilitated re-appraisal of the fragment upon re-entry into the poem’s first line and relatively unaffected confirmation of the prioritized solution in the second reading of the phrase lines. On this depiction, the time savings with L.1-cut haiku may be due to the cut marker emphasizing the cut and reinforcing an FG mode of reading, which in turn would facilitate the transition to, and taking-in of, the phrase lines and thus the integration of two images.

(5) **The cut marker – although foremost a salient structural feature – may also provide cues to meaning.** The reader may be more receptive to these cues when reading in FG mode, and taking these cues into account can facilitate global meaning construction. Our tentative analysis of specific-marker effects (in juxtaposition haiku) revealed that ellipsis markers produced benefits in terms of total processing time for L.1-cut haiku (mainly due to reducing the cut effect in the second/third reading pass), and only moderate costs for L.2-cut haiku (associated with an increased cut effect mainly in the first pass). By contrast, dash markers made little difference relative to no markers for L.1-cut haiku, but produced a marked cost (associated with a substantially increased cut effect originating mainly in the first pass) for L.2-cut haiku. Overall, this would suggest that ellipsis markers are more facilitative to meaning construction/resolution than dash markers. While dash markers emphasize a break or pause before the introduction of unexpected material (thus tending to slow down reading), ellipsis markers hint at something that is left unsaid (but implied) and so might trigger active ‘generation’ processes (i.e., working out what is implied) that may ultimately help bridge the gap and promote understanding. While a (post-hoc) analysis of subjective responses supports this view (i.e., understanding achieved with ellipsis vs. dash markers: 2.75 vs. 2.45, BF10=27.28), this interpretation needs further corroboration using a larger sample of poems (including context–action haiku); also a larger variety of cut markers (than just ellipses and dashes) may need to be explored in future work.

(6) **Subjective ratings reflect mainly the ‘difficulty’ of a haiku**. Taken together, juxtaposition haiku and L.2-cut haiku were consistently experienced as ‘harder to read’, evidenced by less ‘understanding achieved’ and less experience of ‘aha’, compared to context-action haiku (as well as one-image haiku). This corroborates an interpretation of the cut effects, which were more pronounced for juxtaposition haiku and haiku with a cut at the end of line 2, in terms of the ‘difficulty’ of resolving the meaning of the haiku. To some extent, ‘difficulty’ also impacts ‘liking’ (haiku experienced as most ‘difficult’, i.e., L.2-cut juxtaposition, were liked least), though not necessarily ‘aesthetic appeal’ (here, context-action haiku had greater subjective appeal when the cut occurred at the end of line 2). Ratings of surprise and emotionality showed no marked, or easily interpretable, differences among the various haiku conditions, that is: either the various conditions were well equated in terms of these ‘constructs’, or these measures are less sensitive in picking up differential subjective experiences among the various conditions. Of note, the approach adopted here to the analysis of the subjective ratings was the same as that applied to the eye-movement measures. A different approach would be to examine the eye-movement patterns as a function of the subjective ratings (e.g., do eye-movement patterns differ as a function of, say, the rated ‘insight’ potential of a poem). Such a more poem-centered analysis approach (using linear-mixed models) has to be deferred to a future study.

### Conclusion and outlook

An important issue in (literary) reading research concerns the way readers process ambiguous texts with regard to their constitutive BG–FG components/images and with regard to which type of information they use in deciding on a given interpretation. In the present study, we addressed these questions using a short form of poetry, namely, normative English Language Haiku (ELH), as paradigmatic material for studying meaning construction. The results demonstrate that, out of the elements created by the poet (fragment, phrase) and skillfully placed into a dynamic relationship using such techniques as the juxtaposition of images and the cut, the reader is made to recreate in her/his mind the pattern intended by the poet, more precisely, one pattern from within the poem’s larger meaning potential. This interactive process between the poem and the reader gives rise to a characteristic pattern of eye movements and fixations across the text, indicative of the type of haiku (context–action vs. juxtaposition), the cut marker (present vs. absent), and the position of the cut (after L.1 vs. after L.2).

In fact, semantic-conceptual and formal-structural poem properties may come to interact in ELH reading in generating the specific eye-movement patterns reflecting ‘strategies’ of meaning resolution. Semantic properties of (juxtaposition vs. context–action type) ELH may recruit relatively early ‘surprise/conflict’ detection/resolution processes. This more content-driven process would be complemented by a more surface-based process that ‘looks out’ for cut markers in the text. If such a marker is encountered, meaning resolution processes are biased towards the post-cut line/s (lines 2 and 3 in L.1-cut haiku, line 3 in L.2-cut haiku). This would be consistent with the notion that markers act as prompts for meaning wrap-up (42).

However, whether the cut marker will have a net facilitatory effect on meaning construction or a more detrimental effect appears to depend on the reading mode in which it is encountered: If encountered in FG mode (evoked by the fragment in line 1 of the haiku), it can be immediately used, and integrated, in meaning resolution processes. By contrast, if encountered in BG mode (fostered by the phrase in lines 1 and 2), its effect may be more disruptive, so that extended processing is required on subsequent passes to achieve a coherent interpretation. This would also explain why, with L.2-cut haiku, the cut marker effect appeared overall more marked with juxtaposition haiku.

Given the very pronounced cut effects described above, it would be interesting to compare, in future work, the reading of normative, three-line haiku with that of one-line haiku (*monoku*). In contrast to the three-line haiku examined exclusively in the present study, in which the cut is typically clear (even without explicit marker), in monoku, the position of the cut is often ambiguous. That is, loading the poem with multiple ambiguities is intentional: the best monoku characteristic of the form is designed to permit, and induce, play with different segmentations of the poem’s elements and thus different (re-)constructions of the haiku’s meaning. An additional technique of interest in monoku is the omission of the fragment from the poem: rather than juxtaposing two images in a tense relationship, in monoku “a single image is extended or elaborated into a second context, often implied” (32, 68) – a technique which complicates the reader’s task of meaning analysis and construction and renders monoku a particularly valuable comparative form to the normative haiku composed of fragment and phrase. Thus, arguably, examining how this potential for multiple meanings is reflected in the reading eye movements can be best assessed using one-line haiku.

Another interesting and important question for future research would be whether the differences in syntactic/semantic- and structure/surface-based processes (including their interactions) demonstrated here for the cut effect in the reading of ELH are also expressed in other measures, besides the eye-movement activity investigated here. While eye-movement measures are potentially informative of key mental processes going on while reading haiku, they would need to be augmented by ‘brain’ measures to achieve a more complete, and complementary, ‘neuro-cognitive’ picture of these processes. Since we obtained a record of participants’ EEG while they were reading the haiku, in the next step, informed by the timing of our signature oculomotor patterns, we will look for neural correlates of the proposed cognitive and aesthetic events during reading, such as oscillatory activity related to insight (69) or evoked responses related to violations of semantic context (N400: see, 70). Apart from validating our speculations on the presence of these events, the amplitude of the respective neuronal markers might provide additional insights into the modulations induced by the features examined (haiku type, cut position, presence and type of cut marker), and their latencies might provide a more fine-grained picture on the temporal dynamics of these events and their order of occurrence (71). This work is in progress.

### Ethics and Conflict of Interest

The author(s) declare(s) that the contents of the article are in agreement with the ethics described in http://biblio.unibe.ch/portale/elibrary/BOP/jemr/ethics.html and that there is no conflict of interest regarding the publication of this paper.

## Footnotes

Following Jacobs and colleagues (e.g., Schrott R & Jacobs AM, München: Carl Hanser, 2011), we are relating here to the definition of fore-grounding prevalent in the formalist/structuralist tradition in literary theory. On this definition, what is foreground-ed is unfamiliar – it ‘stands out’ from its surroundings by being unexpected: by possessing “salience by surpris-al” or “novelty” – and results in de-familiarizing and de-automatizing effects. In contrast, other notions of fore-grounding assume that what is salient is what is easily accessible from memory (“salience by entrenchment”; e.g., Schmid H-J & Günther F, Front. Psychol., 2016) or what is assigned prominence in a sentence, usually via the strategic use of information structural means (e.g., van Dijk TA & Kintsch W, New York: Academic Press, 1983). As the present study was not designed to delineate the notion(s) of foregrounding at work in the reading of haiku, this issue must be deferred to future work.

## Appendix

### Analyses of reading material

To test our hypotheses conclusively, we had to rule out that any effect patterns obtained (differential oculomotor reading dynamics between haiku types, cut placements, presence/absence of cut markers) were systematically influenced by (uncontrolled) variations of the poems’ general, ‘haiku-unspecific’ language characteristics. Therefore, and inspired by the reading literature referred to in the following, all poems were analyzed for, and eventually balanced with respect to, the following set of parameters:

1. **Item-length-related parameters**: the following features were counted (per poem line):
  - graphemes/letters
  - syllables
  - morphemes
  - (orthographic) words
  - phrases (high-level phrases, i.e. phrase types that could function as syntactic constituents)
2. **Frequency-related parameters**: all poems were checked for the occurrence of
  - low-frequency words
  - low-frequency (two-word) collocations

Low-frequency occurrence is defined here as less than one token by million words in the British National Corpus (72); frequency data were calculated using Sketchengine (73); information on effects of word frequency on eye movements during reading can be found, e.g., in (74, 75, 76).

3. **Categorial and constructional variables** (determined by line):
  - ratio of content to function words (and thus to words which tend to be skipped by readers; e.g., 77, 78, 79)
  - (variation in) position and form of realization (finite, infinite, ellipted) of the verb (as the central valency carrier and thus determinant of sentence structure; e.g., 80)
  - (frequency and context of) occurrence of phoric elements like pronouns or definite determiners (the identification of whose antecedents has been reported to result in longer fixation durations and/or regressive saccadic movements; e.g., 80, 81, 82).
4. **Stylistic variables** (counted by line): (frequency of) occurrence of
  - unusual syntactic patterns (i.e., word order other than SVO)
  - potentially attention-attracting stylistic features like
  - alliterations
  - (sentence- and phrase-internal) enjambments (36).

To rule out potentially confounding effects of the (unbalanced) occurrence of these features in the haiku set used in the experiment, it was necessary to test for the absence of differences in these linguistic variables among our eight two-image haiku (haiku type x cut position x cut marker) conditions and between these and the one-image haiku condition. Because nonsignificant results could only be interpreted as absence of evidence, we used Bayes Factors, which can also be interpreted as evidence of absence if they are sufficiently small (57, 83). We computed Bayes Factors using Bayesian linear models, equivalent to an analysis-of-variance (ANOVA) design. For the analyses of the 13 formal language characteristics (i.e., linguistic analysis), we computed the Bayes Factor for the effect of the factor poem line (since numbers of words were, on average, highest in the central poem line, e.g., number of words: line 1: 2.0 words; line 2: 3.38 words; line 3: 2.50 words) and compared this ‘line-effect only’ model to the effects/models arising from our experimental factors (haiku type, cut placement, cut marker) or combinations/interactions of these. This led to a total of seven comparisons (three main effects, four interactions) between the line-only and our experimental-factor models for each language parameter coded (see above). Thus, for the analysis of what we defined as ‘general’ linguistic variables, a given (high) Bayes Factor would indicate a better fit of a given experimental-factor model relative to the line-factor model. In other words, high Bayes Factors indicate that differences in language features are associated with our experimental manipulations, rather than (general, haiku-unspecific) differences in these features across the three poem lines.

Two sets of analyses were conducted to assess whether our two-image ELH, in the experimental – haiku type, cut placement, and cut marker – conditions, differed with regard to a total of 13 formal language components (analysis set 1) and whether there were differences in these components between two-image (context–action and juxtaposition) and one-image haiku (analysis set 2).

Analysis 1, of the two-image ELH presented during reading, failed to reveal any systematic difference in any of these general, haiku-unspecific linguistic variables as a function of poem type, cut placement, and cut marker. The median BF10 across all 7 possible effects (3 x main effects/4 x interactions) and 13 linguistic variables (number of letters/word, number of syllables/word, etc.) was .24, with a 25th (75th) quantile of .02 (.31).

Analysis 2 compared two-image with one-image ELH across the 13 linguistic parameters. Since the number of two-image haiku exceeded that of one image haiku presented for reading (64 vs. 8), we randomly selected a subset of 8 two-image haiku and compared this set against the 8 one-image haiku presented in the reading phase. There were no substantial effects in any of the linguistic parameters coded, median BF10=.58; 25th quantile: .48; 75th quantile: .59. Given the absence of any such differences, both between the two-image ELH in the various experimental conditions as well as between the two-image and one-image ELH, it is unlikely that any of the effects reported below on readers’ eye-movement behavior are mainly/primarily attributable to differences in general, haiku-unspecific language variables.
